# In Vitro Cartilage Regeneration with a Three-Dimensional Polyglycolic Acid (PGA) Implant in a Bovine Cartilage Punch Model

**DOI:** 10.3390/ijms222111769

**Published:** 2021-10-29

**Authors:** Victoria Horbert, Long Xin, Peter Föhr, René Huber, Rainer H. Burgkart, Raimund W. Kinne

**Affiliations:** 1 Experimental Rheumatology Unit, Orthopedic Professorship, Jena University Hospital, Waldkliniken Eisenberg GmbH, 07607 Eisenberg, Germany; victoria.horbert@med.uni-jena.de (V.H.); xinlonghz@gmail.com (L.X.); 2 Department of Orthopedics, Tongde Hospital of Zhejiang Province, Hangzhou 310012, China; 3 Biomechanics Laboratory, Chair of Orthopedics and Sport Orthopedics, Technische Universität München, 81675 Munich, Germany; foehr@tum.de (P.F.); burgkart@tum.de (R.H.B.); 4 Institute of Clinical Chemistry, Hannover Medical School, 30625 Hannover, Germany; huber.rene@mh-hannover.de

**Keywords:** bovine cartilage punch model, polyglycolic acid (PGA), articular cartilage regeneration, push-out test for implants

## Abstract

Resorbable polyglycolic acid (PGA) chondrocyte grafts are clinically established for human articular cartilage defects. Long-term implant performance was addressed in a standardized in vitro model. PGA implants (+/− bovine chondrocytes) were placed inside cartilage rings punched out of bovine femoral trochleas (outer Ø 6 mm; inner defect Ø 2 mm) and cultured for 84 days (12 weeks). Cartilage/PGA hybrids were subsequently analyzed by histology (hematoxylin/eosin; safranin O), immunohistochemistry (aggrecan, collagens 1 and 2), protein assays, quantitative real-time polymerase chain reactions, and implant push-out force measurements. Cartilage/PGA hybrids remained vital with intact matrix until 12 weeks, limited loss of proteoglycans from “host” cartilage or cartilage–PGA interface, and progressively diminishing release of proteoglycans into the supernatant. By contrast, the collagen 2 content in cartilage and cartilage–PGA interface remained approximately constant during culture (with only little collagen 1). Both implants (+/− cells) displayed implant colonization and progressively increased aggrecan and collagen 2 mRNA, but significantly decreased push-out forces over time. Cell-loaded PGA showed significantly accelerated cell colonization and significantly extended deposition of aggrecan. Augmented chondrogenic differentiation in PGA and cartilage/PGA-interface for up to 84 days suggests initial cartilage regeneration. Due to the PGA resorbability, however, the model exhibits limitations in assessing the “lateral implant bonding”.

## 1. Introduction

Tissue engineering (TE) has become a major field of research in regenerative medicine [1], including areas such as bone [2], skin [3], muscle [4], cancer [5], and cardiac TE [6]. Suitable biomaterials of natural or synthetic origin have central importance for the success of regenerative TE and must thus fulfill pivotal pre-requisites concerning biocompatibility, biodegradability (generally desired), morphology, pore size/porosity, and mechanical strength [7]. This allows the tailoring of the features of the particular biomaterial to the needs of the respective TE approach, for example, in view of the increasingly aging populations in the industrialized countries.

Synthetic biopolymer-based scaffold materials, e.g., polyglycolic acid (PGA), polylactid acid (PLA), or their co-polymer polylactic-co-glycolic acid (PLGA), generally combine several advantages over natural biomaterials such as high batch-to-batch consistency, favorable processability and solubility blocking, absence of contaminating pyrogens and pathogens, optimized and tailored material properties, and low cost [7]. Synthetic biomaterials have thus found successful clinical application as suture material, stents, bone fixation devices, wound meshes, meniscus or cartilage repair devices, or artificial dura [7].

Cartilage injury due to trauma or degeneration affecting either the full or partial thickness of the knee articular cartilage occurs very frequently, often leads to progressive cartilage decay, and culminates in osteoarthritis. Cartilage repair can be sought by different methods including autologous chondrocyte implantation (ACI), microfracture, and osteoarticular transfer (OATS)/mosaicplasty, and some of these procedures have shown promising clinical results for initial cartilage regeneration [8,9]. The quality of the repaired cartilage, however, is still suboptimal, and the tissue often shows features of fibrous cartilage, strongly reduced mechanical strength, and a higher permeability than native cartilage [10,11]. ACI techniques of the second generation, e.g., matrix-assisted chondrocyte implantation/transplantation (m-ACI/MACT), have recently been developed for clinical practice, with the primary aim to support stable and extended bonding of the transferred cells to the edges of the injured cartilage [12,13,14,15]. For this purpose, implants such as membranes composed of type I/III collagen have been used [16,17,18], which reduce operation duration and surgical trauma and avoid complications related to the application of periosteum (e.g., graft overgrowth).

A commercial m-ACI product (BioSeed-C^®^), an autologous, three-dimensional, cell-loaded, resorbable cartilage transplant, has been applied in Europe with some clinical success [19,20,21,22,23]. One study in the large animal sheep has recently demonstrated that even a cell-free PGA-hyaluronan implant (chondrotissue^®^) combined with microfracturing of the subchondral plate yields cartilage repair equivalent to that observed after implanting cell-seeded PGA [24]. The original choice of PGA as a biomaterial was based on: (i) long-term experience and use as a medical device for, e.g., suture material, osteofixation, and cartilage repair [2,7,25,26]; (ii) its character as a non-natural, synthetic product, avoiding problems such as rejection for religious reasons and virus or prion contamination but allowing easy melting, processing, or spinning for manufacturing [7]; (iii) its complete resorption in vivo within 3 months [27], following hydrolytic breakdown into natural degradation products [7]. In this context, potential induction of inflammation and osteolysis by breakdown products of such synthetic biopolymers may be more of a problem for bulk screw and osteofixation biomaterials than for scaffolds and sutures [1,2,7,25,28]. Additionally, the relative suitability of PGA/PLGA versus alginate, hyaluronic acid, or chitosan, which are similar to and/or interact with local glycosaminoglycans for cartilage regeneration [1], should be assessed by ‘contemplating both potential advantages and disadvantages of each technique’ [7] and by optimizing the match between ‘the final product properties…and the needs of specific tissues to be regenerated’ [7].

However, there are at present no in vitro analyses examining the cellular or molecular mechanisms underlying cartilage repair when using PGA. This study thus aimed at investigating the behavior of the resorbable, three-dimensional PGA implant in a previously established bovine cartilage punch model for the examination of different cartilage implants [29,30,31] and to answer the question whether the experimental results mirror the clinical performance of the PGA. The addressed hypotheses were as follows: (i) the model allows detailed analysis of the underlying in vitro cartilage repair in and around the PGA implant; (ii) PGA supports in vitro cartilage regeneration (through colonization and matrix formation) on the basis of its physicochemical and molecular features.

Bovine “host” cartilage and PGA implants retained their vitality with preserved matrix and limited proteoglycan loss throughout culture until 84 days (12 weeks). PGA implants (+/− cells) favored initial cell colonization and cartilage differentiation/repair with an advantage of cell-loaded PGA concerning earlier colonization and more extended deposition of aggrecan.

## 2. Results

### 2.1. Cell-Free PGA

#### 2.1.1. Morphological Features

**Figure 1:**
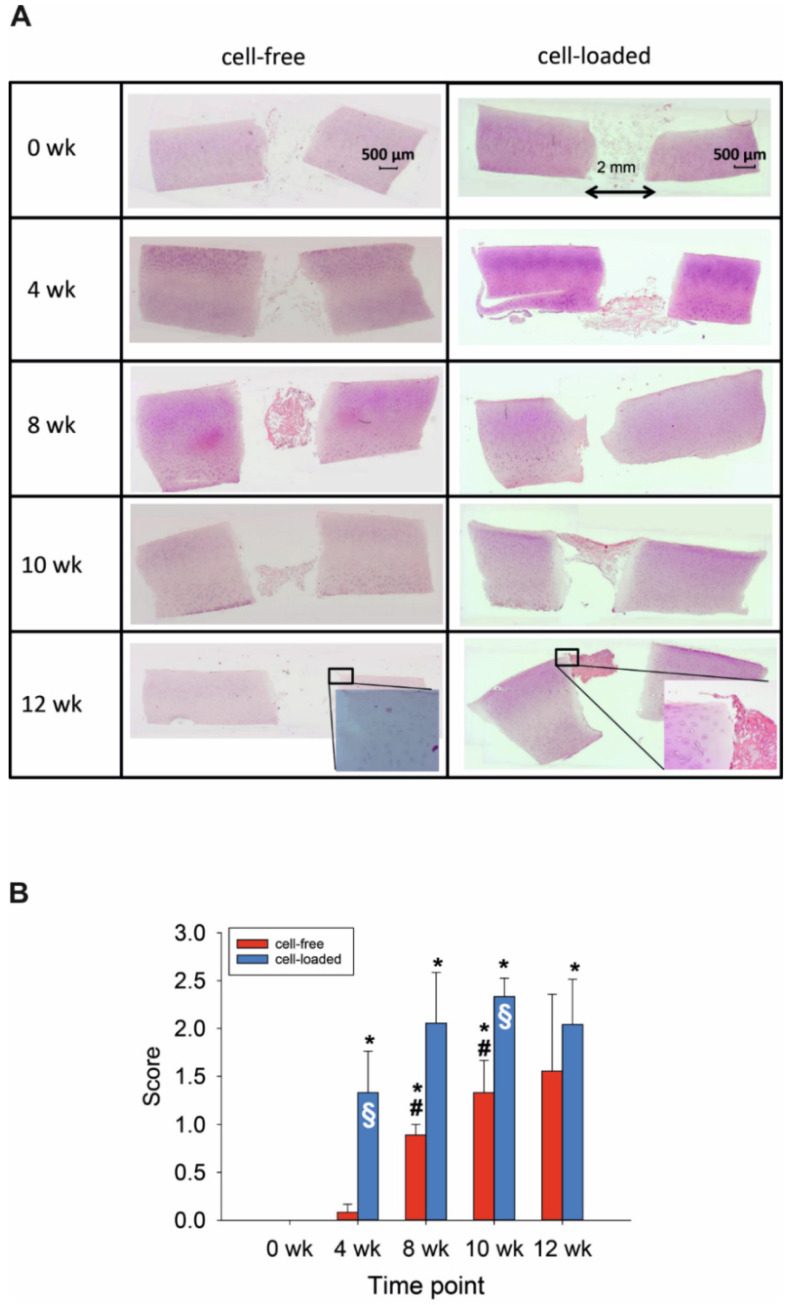
(**A**) Hematoxylin/eosin (HE) staining of the cartilage/PGA hybrids (+/− cells) during in vitro culture. (**B**) Semiquantitation of PGA colonization (+/− cells). Degree of migration: 0 = implant without cells; 1 = single adherent cells; 2 = several adherent cells; 3 = cell-layer on implant; means +/− standard error of the mean; *p* ≤ 0.05 versus: * 0 weeks; or # 4 weeks; § versus cell-free.

Resorbable, three-dimensional PGA maintained the lateral contact to the cylindrical defect for at least 4 weeks (Figure 1A). Despite culturing for up to 84 days, resident chondrocytes remained vital (without signs of pathological changes; positive staining of cell nuclei), suggesting highly favorable in vitro conditions (Figure 1A). The integrity of the cartilaginous matrix was largely unaltered over time (Figure 1A). However, possibly as a response to an initial mechanical trauma by the biopsy punch, cartilage areas close to the edge of the defect contained proliferation-induced cell clusters (Figure 1A; see insert for 12 weeks). The first clear colonization of the initially cell-free PGA was observed after 8 weeks (Figure 1A,B), with a significant increase from 0 and 4 weeks to 8 and 10 weeks (Figure 1B).

#### 2.1.2. Content of Cartilage Matrix Proteins (Tissue)

**Figure 2:**
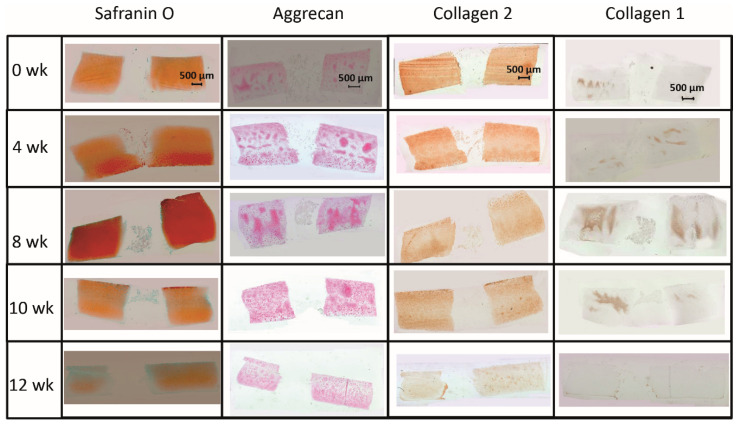
(Immuno)staining of PGA (cell-free) during in vitro culture.

**Figure 3:**
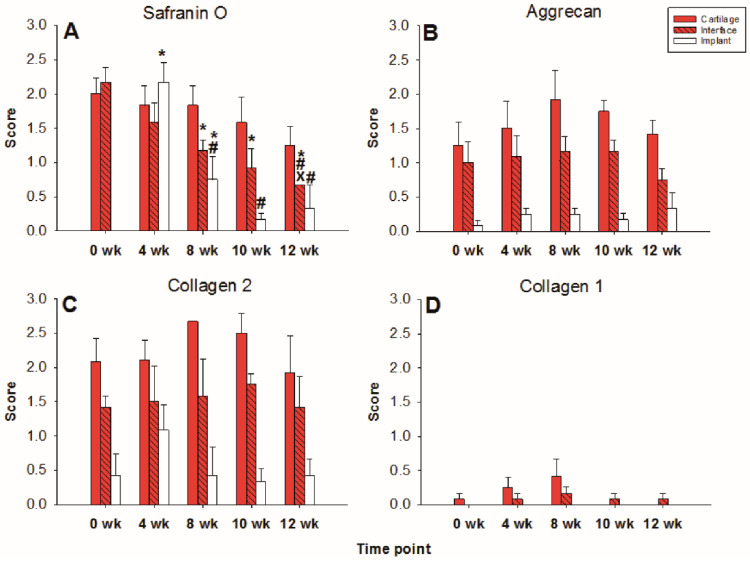
Semiquantitation of the (immuno)staining for Safranin O (**A**), aggrecan (**B**), collagen 2 (**C**), and collagen 1 (**D**) in “host” cartilage, cartilage–implant interface, and PGA implant (cell-free) after different periods of in vitro culture. Score: 0 = no staining, 1 = weak staining, 2 = moderate staining, 3 = strong staining; means +/− standard error (SEM) of the mean; symbols show *p* ≤ 0.05 versus: * 0 weeks; # 4 weeks; or X 8 weeks.

The limited decrease of **safranin O staining** score during culture in surrounding cartilage rings (from 2.0 for fresh cartilage to 1.25 at 12 weeks; Figure 2 and Figure 3A) was not significant, which indicated a conserved integrity of the cartilage matrix and a limited liberation of proteoglycans over time. The safranin O score in the cartilage–implant interface also decreased (from 2.1 to 0.6), with statistical significance versus 0 weeks for the 8, 10, and 12 week values and versus 4 and 8 weeks for the 12 week values (Figure 2 and Figure 3A). In the implant, there was strong positive staining already at 4 weeks, with a significant decrease thereafter (Figure 2 and Figure 3A; *p* ≤ 0.05 versus 0 weeks for 4 and 8 weeks; *p* ≤ 0.05 versus 4 weeks for 8, 10, and 12 weeks). This indicates an at least transient proteoglycan deposition into the implant.

The **aggrecan immunostaining** score in the surrounding cartilage ring and cartilage–implant interface was largely stable during culture (stable scores ranging from 0.8 to 1.9; Figure 2 and Figure 3B), also indicating a limited proteoglycan loss over time. The score in the PGA first reached substantial levels at 4 weeks, with a subsequent plateau (stable scores ranging from 0.1 to 0.3; Figure 2 and Figure 3B), certifying proteoglycan deposition into the implant.

The **collagen 2 immunostaining** score for this matrix protein during culture was also largely stable in surrounding cartilage (from 2.0 to 2.6; intermediate peak at 8 weeks), cartilage–implant interface (between from 1.4 to 1.7), and PGA (from 0.5 to 1.1; intermediate peak at 4 weeks), without any time dependency (Figure 2 and Figure 3C), again underlining a preserved matrix integrity.

In the case of **collagen 1**, very little immunostaining occurred in the different compartments (scores from 0 to 0.4); there was no time dependency (Figure 2 and Figure 3D).

#### 2.1.3. Concentration of Proteoglycans in Tissue and Supernatant (Dimethylmethylene Blue, DMB-Test)

The decrease of the glycosaminoglycans (GAG) content during culture in the cartilage ring was limited and not significant (from 4389 µg/mL for fresh cartilage to 1831 µg/mL at 12 weeks; Supplementary Figure S1A), which confirms the findings of both safranin O scores and aggrecan immunostaining. Chondrocytes migrated onto the cartilage surface in general showed approximately 10-fold lower GAG levels, with a numerical decrease until 10 weeks (from 370 µg/mL for freshly isolated cartilage to 187 µg/mL at 10 weeks) and a subsequent increase to 702 µg/mL until 12 weeks (Supplementary Figure S1A). The GAG levels in the supernatant also showed a very limited, non-significant decline during culture (from 124 µg/mL at 4 weeks to 105 µg/mL at 10 weeks and 120 µg/mL at 12 weeks; Supplementary Figure S1A), in parallel to an almost unchanged liberation of aggrecan into the supernatant over time (enzyme-linked immunosorbent assay (ELISA): from 19 ng/mL at 4 weeks to 21 ng/mL at 12 weeks; not shown).

#### 2.1.4. Collagen 2 and 1 Content of Supernatant (ELISA)

The decrease of collagen 2 and collagen 1 liberation into the supernatant during culture was also limited and non-significant (Collagen 2: from 996 ng/mL at 4 weeks to 63 ng/mL at 12 weeks; Supplementary Figure S1C; Collagen 1: from 120 ng/mL at 4 weeks to an intermediate peak of 212 ng/mL at 8 weeks with a decrease thereafter; data not shown).

#### 2.1.5. mRNA Levels for Cartilage Matrix Proteins (Quantitative Real-Time Polymerase Chain Reaction, qRT-PCR)

**Figure 4:**
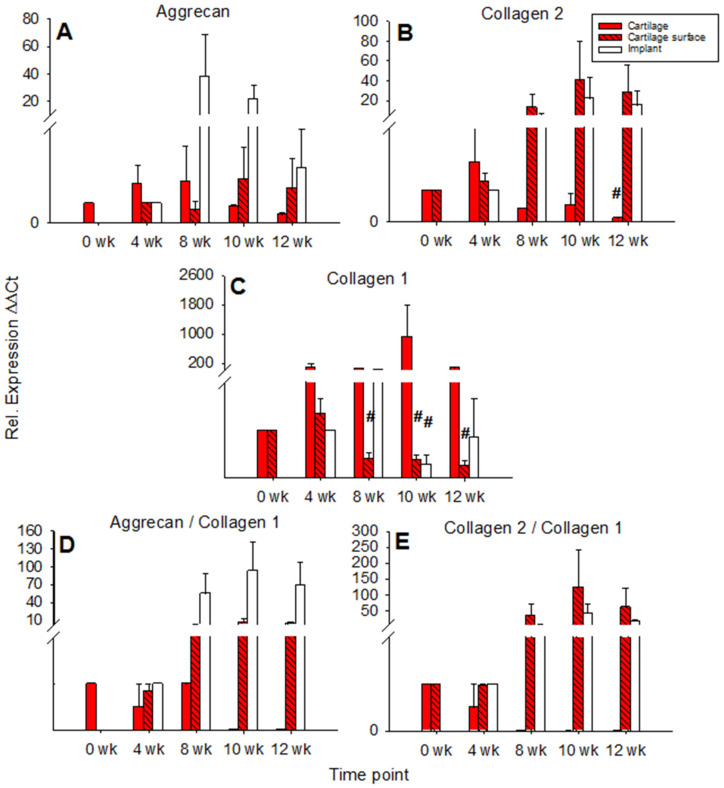
Real time PCR analyses for cartilage matrix proteins (cell-free PGA). mRNA levels for aggrecan (**A**), collagen 2 (**B**), collagen 1 (**C**), aggrecan/collagen 1 ratio (**D**), and collagen 2/collagen 1 ratio (**E**) were analyzed before and after 4, 8, 10, and 12 weeks of culture; relative gene expression of the chondrocytes in the cartilage matrix (cartilage), on the cartilage surface (cartilage surface), and on/in the PGA (implant); means +/− SEM; # = *p* ≤ 0.05 versus 4 weeks.

Aggrecan expression in cartilage ring and cartilage surface cells was not significantly changed over time with an intermediate peak at 4 and 8 weeks for the cartilage ring (max. 2-fold in comparison to 0 weeks) and a later peak at 10 and 12 weeks for the surface cells (also max. 2-fold in comparison to 4 weeks). By contrast, aggrecan expression in the PGA showed an intermediate, solid peak at 8 weeks (max. 38-fold) and a subsequent decrease (Figure 4A).

mRNA levels for collagen 2 in the cartilage ring showed a significant decrease during culture (*p* ≤ 0.05 for 12 weeks versus 4 weeks), while on the other hand, the mRNA levels in cartilage surface cells and PGA increased to intermediate peaks at 10 weeks of 40-fold and 22-fold, respectively, and subsequently slightly declined (Figure 4B). mRNA levels for collagen 1 in the cartilage increased to an intermediate peak at 10 weeks and thereafter decreased again. Cartilage surface cells and PGA also reached intermediate peaks of collagen 1 mRNA at 4 and 8 weeks, respectively, with a significant decrease thereafter (surface cells: *p* ≤ 0.05 for 8, 10, and 12 weeks versus 4 weeks; PGA: *p* ≤ 0.05 for 10 weeks versus 4 weeks; Figure 4C). The aggrecan/collagen 1 ratio in the cartilage ring decreased over time, a substantial, long-lasting increase of the aggrecan/collagen 1 ratio was noted in cells on the cartilage surface (max. 7-fold) and PGA implant (92-fold; both at 10 weeks; Figure 4D). Similarly, the collagen 2/collagen 1 ratio decreased over time in the cartilage ring but highly increased in cartilage surface cells (122-fold) and PGA (41-fold; both at 10 weeks; Figure 4E).

### 2.2. Cell-Loaded PGA

#### 2.2.1. Morphological Features

The findings for cell-loaded PGA were in general similar to those for cell-free PGA, with vital morphology of resident cartilage during in vitro culture and largely preserved matrix integrity, but with bilateral contact to the cylindrical defect until at least 10 weeks (Figure 1). Additionally, there was an initial presence or early colonization with chondrocytes of cell-loaded PGA implants already after 4 weeks (Figure 1A,B); this parameter was significantly higher comparing 0 weeks to all later points (Figure 1B). This resulted in a significantly earlier and long-lastingly higher cell colonization for cell-loaded PGA implants than for initially cell-free PGA (*p* ≤ 0.05 at 4 and 10 weeks; Figure 1B).

#### 2.2.2. Content of Cartilage Matrix Proteins (Tissue)

**Figure 5:**
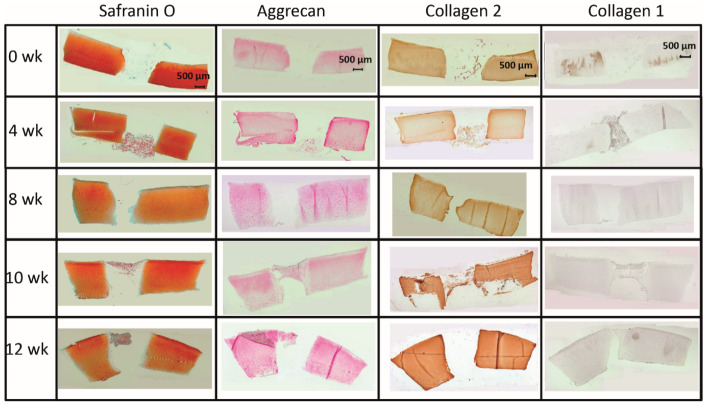
(Immuno)staining of PGA (cell-loaded) during in vitro culture.

**Figure 6:**
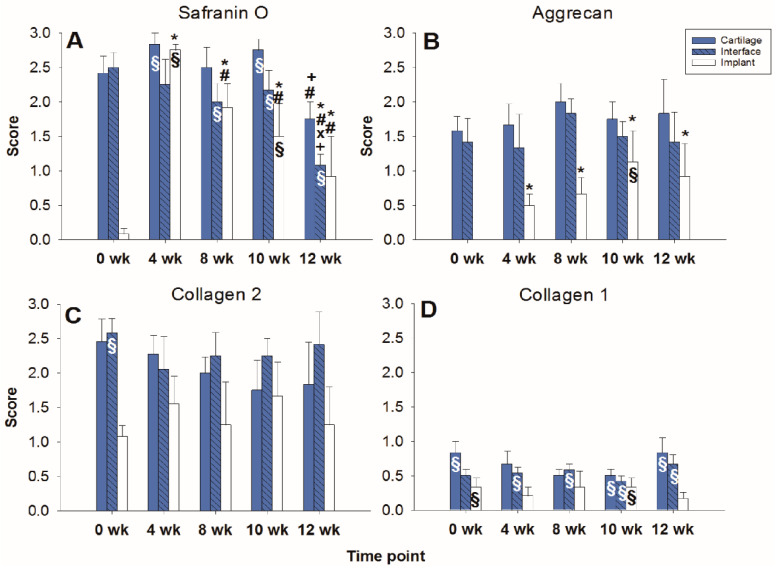
Semiquantitation of the (immuno)staining for Safranin O (**A**), aggrecan (**B**), collagen 2 (**C**), and collagen 1 (**D**) in “host” cartilage, cartilage–implant interface, and PGA implant (cell-loaded) after different periods of in vitro culture. Score: 0 = no staining, 1 = weak staining, 2 = moderate staining, 3 = strong staining; means +/− standard error (SEM) of the mean; symbols show *p* ≤ 0.05 versus: * 0 weeks; # 4 weeks; x 8 weeks; or + 10 weeks; § versus cell-free.

In the cell-loaded group, the **safranin O staining score** showed a limited but significant decline of over time in the cartilage ring (from 2.4 for the fresh cartilage to 1.7 at 12 weeks; *p* ≤ 0.05 for 12 versus 4 and 10 weeks; Figure 5 and Figure 6A) and a decrease of staining (from 2.5 to 1.0) in the cartilage–implant interface, which was statistically significant for the 12 week value compared to 0, 4, 8, and 10 weeks (Figure 5 and Figure 6A). In PGA, a remarkable increase of the staining intensity to very high levels was noticed at 4 weeks, with a subsequent significant decrease to moderate levels (*p* ≤ 0.05 higher than 0 weeks for all different time points; *p* ≤ 0.05 lower than 4 weeks for the 8, 10, and 12 week time point; Figure 5 and Figure 6A). Concerning the comparison with cell-free implants, the score for cell-loaded PGA at 4 and 10 weeks was significantly larger compared to cell-free PGA (compare Figure 3A and Figure 6A).

As for safranin O, a largely stable **aggrecan immunostaining** was noted in cartilage ring and interface during in vitro culture (stable scores ranging from 1.3 to 2.0; Figure 5 and Figure 6B). In PGA, aggrecan staining was first detectable at 4 weeks with a significant increase throughout in vitro culture (*p* ≤ 0.05 versus 0 weeks for 4, 8, 10, and 12 weeks; Figure 5 and Figure 6B). As for safranin O, the score for cell-loaded PGA at 10 weeks was significantly larger compared to cell-free PGA (compare Figure 3B and Figure 6B).

A largely constant **immunostaining for collagen 2** was noticed during culture in cartilage ring (scores from 1.7 to 2.4), interface (from 2.0 to 2.6), and PGA (from 1.0 to 1.6); the scores were not significantly different among time points (Figure 5 and Figure 6C).

In the case of **collagen 1**, very little immunostaining (scores largely from 0.2 to 0.8) was noticed in the cartilage ring, interface, and PGA (Figure 5 and Figure 6D). Nevertheless, the score at 0 and 10 weeks for cell-loaded PGA was significantly larger compared to cell-free PGA (compare Figure 3D and Figure 6D).

#### 2.2.3. Proteoglycan Content in Tissue Extracts and Culture Supernatant (DMB-Test)

For cell-loaded PGA, the decline of the GAG concentration in the cartilage ring during culture (from 4525 µg/mL for fresh cartilage to 3853 µg/mL at 12 weeks, including an intermediate 4-week peak of 4784 µg/mL; Supplementary Figure S1B) was limited and did not reach statistical significance. The chondrocytes migrated onto the cartilage surface again showed an approximately 10-fold lower GAG content than the cartilage ring, with a numerical increase during culture (from 185 µg/mL for fresh cartilage to 1122 µg/mL at 12 weeks; Supplementary Figure S1B). There were only marginal GAG content differences between cell-free and cell-loaded PGA (compare Supplementary Figure S1A,B).

The limited decline of the GAG concentration in the supernatant during culture (from 136 µg/mL at 4 weeks to 104 µg/mL at 10 weeks, with a slight increase to 113 µg/mL at 12 weeks; Supplementary Figure S1B) reached statistical significance, as also supported by the limited, non-significant decline of the aggrecan liberation into the supernatant during culture (ELISA; from 17 ng/mL at 4 weeks to 1 ng/mL at 12 weeks; data not shown).

#### 2.2.4. Collagen 2 and 1 Content of Culture Supernatant (ELISA)

The collagen 2 liberation into the supernatant was significantly decreased and the collagen 1 release non-significantly increased (collagen 2: from 531 ng/mL at 4 weeks to 130 ng/mL at 12 weeks; *p* ≤ 0.05 versus 4 weeks for 10 and 12 weeks; Supplementary Figure S1D; collagen 1: from 115 ng/mL at 4 weeks to 191 ng/mL at 10 weeks with an intermediate peak at 8 weeks; not shown).

#### 2.2.5. mRNA Levels for Cartilage Matrix Proteins (RT-PCR)

**Figure 7:**
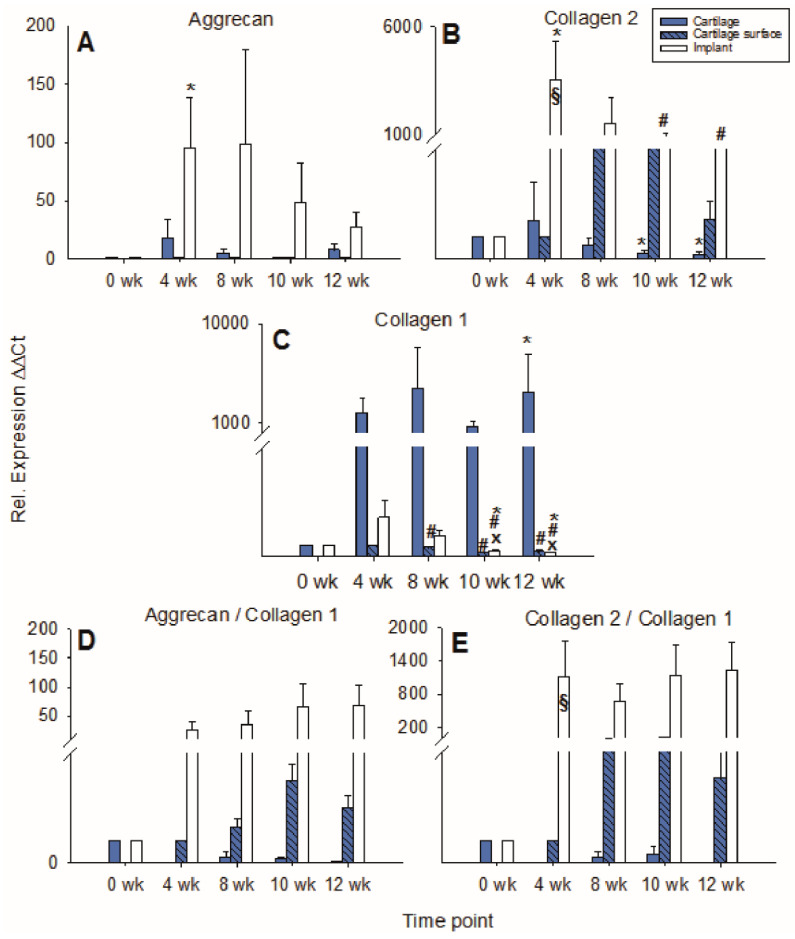
Real-time PCR analyses for cartilage matrix proteins (cell-loaded implants). mRNA levels for aggrecan (**A**), collagen 2 (**B**), collagen 1 (**C**), aggrecan/collagen 1 ratio (**D**), and collagen 2/collagen 1 ratio (**E**) were analyzed prior to and after 4, 8, 10, and 12 weeks of culture; relative gene expression of the chondrocytes in the matrix of the cartilage (cartilage), on the cartilage surface (cartilage surface), and on/in the PGA (implant); means +/− SEM; symbols show *p* ≤ 0.05 versus: * 0 weeks; # 4 weeks; or x 8 weeks; § versus cell-free.

In cartilage ring and implant, the mRNA expression for aggrecan rose to intermediate peaks at 4 and 8 weeks (17-fold and 98-fold, respectively; **implant**: *p* ≤ 0.05 for 4 weeks vs. 0 weeks) and then decreased again, whereas the mRNA expression for aggrecan in cartilage surface cells remained low and largely stable during culture (Figure 7A).

In the cartilage ring, the collagen 2 expression decreased significantly over time with an intermediate two-fold peak at 4 weeks (*p* ≤ 0.05 for 10 and 12 weeks versus 0 weeks; Figure 7B). By contrast, cartilage surface cells progressively increased their collagen 2 expression until 8 weeks of culture, with a subsequent decrease. Strikingly, in the implant, the collagen 2 expression significantly increased to a transient, substantial 4 week peak (3225-fold) and subsequently diminished but remained at very high levels until 12 weeks (446-fold; *p* ≤ 0.05 for 4 weeks versus 0 weeks, as well as for 10 and 12 weeks versus 4 weeks; Figure 7B). At 4 weeks, in cell-loaded PGA, the collagen 2 expression was significantly larger compared to cell-free PGA (compare Figure 4B and Figure 7B).

The relative gene expression of collagen 1 in the cartilage ring significantly rose during in vitro cultivation (*p* ≤ 0.05 for 12 weeks versus 0 weeks; Figure 7C), while collagen 1 expression in the cartilage surface cells significantly decreased during cell culture (*p* ≤ 0.05 for 8, 10, and 12 weeks versus 4 weeks; Figure 7C). In PGA, collagen 1 expression first increased to a minor, intermediate peak at 4 weeks (4-fold) and subsequently declined significantly (*p* ≤ 0.05 for 10 and 12 weeks versus 0, 4, and 8 weeks; Figure 7C).

The aggrecan/collagen 1 ratio decreased over time in the cartilage ring and substantially increased during culture in cartilage surface cells and PGA (max. 4-fold at 10 weeks and 67-fold at 12 weeks, respectively; Figure 7D). Similarly, the collagen 2/collagen 1 ratio in the cartilage ring decreased over time and increased substantially during cell culture in surface cells and implant (max. 23-fold at 10 weeks and 1226-fold at 12 weeks, respectively; Figure 7E). Notably, at 4 weeks, the collagen 2/collagen 1 ratio in cell-loaded PGA was significantly larger compared to cell-free PGA (compare Figure 4E and Figure 7E).

#### 2.2.6. Push-Out Forces of Cultivated Cartilage/PGA Hybrids (Biomechanical Analyses)

**Figure 8:**
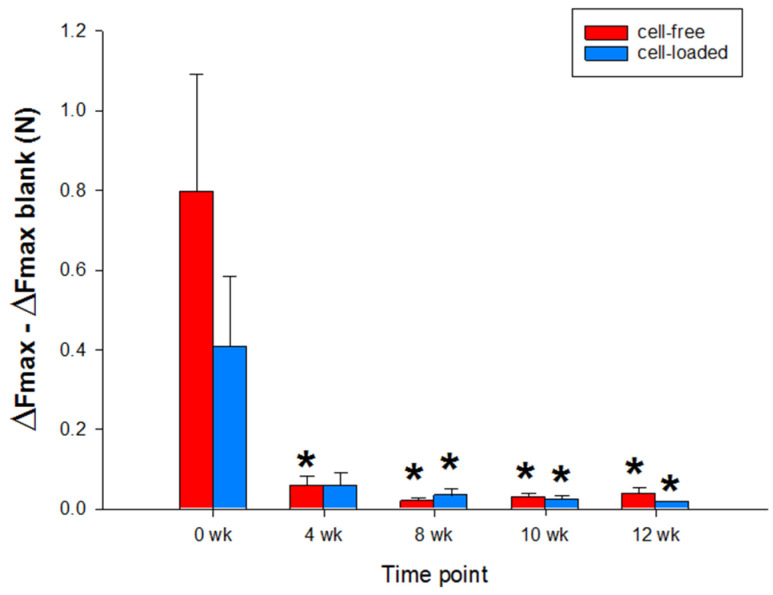
Biomechanical push-out analyses of cartilage/PGA hybrids (cell-free or cell-loaded). Means +/− SEM; symbols show *p* ≤ 0.05 versus * 0 weeks.

Notably, the push-out force of both types of PGA exhibited a continuous decrease over time (cell-free: from 0.796 +/− 0.293 Newton (N) or 975.381 +/− 359.458 Kilopascal (kPa) at 0 weeks to 0.038 +/− 0.008 N or 35.788 +/− 17.471 kPa at 12 weeks; cell-loaded: from 0.407 +/− 0.175 N or 499.106 +/− 215.284 kPa at 0 weeks to 0.017 +/− 0.003 N or 21.232 +/− 4.887 kPa at 12 weeks; *p* ≤ 0.05 for 4, 8, 10, and 12 weeks versus 0 weeks; Figure 8), without significant differences between cell-free and cell-loaded PGA at any time point.

## 3. Discussion

The present study addresses the long-term performance of a three-dimensional PGA implant in a previously established, standardized in vitro bovine cartilage punch model [29,30,31,32,33,34,35,36,37,38,39,40]. The central findings were that: (i) cartilage/PGA hybrids remained vital with an integer cartilage matrix, limited proteoglycan loss in cartilage ring or cartilage–PGA interface, and diminishing release of proteoglycan into the supernatant; and (ii) both types of PGA (cell-free or cell-loaded) displayed cell immigration/colonization and continuously augmented gene expression for aggrecan and collagen 2. Due to the resorbability of the PGA, however, both types of PGA implants exhibited significantly diminished push-out forces over time. On the other hand, cell-loaded PGA showed significantly more rapid cell colonization and significantly more extended aggrecan deposition than cell-free implants. This corresponds well with the described biocompatibility of clinically registered PGA cartilage repair implants (+/− cells) [41,42,43,44].

### 3.1. “Host” Cartilage Ring Performance

As shown previously [29,30,31], the “host” cartilage ring remained stable for extended times of culture, as demonstrated by a low cartilage degeneration and loss of proteoglycan, as well as constant protein content of aggrecan and collagen 2. However, the resident chondrocytes showed some indications for dedifferentiation (especially at later time points), indicating that the present in vitro model principally allows extended in vitro culture with partial parallelity to the in vivo setting, but with limitations at 10 and 12 weeks.

### 3.2. Cartilage Regeneration in the PGA Implants

#### 3.2.1. Cell Colonization of the Implant

Both types of PGA implants showed a colonization of the resorbable implant, which occurred in parallel with chondrocyte emigration in particular from the “host” cartilage surface. This validates the large cytocompatibility of three-dimensional PGA, as previously shown experimentally [32,41,42,43] and clinically [19,20,21,22,23,24,41,45]. Strikingly, cell loading of the PGA implants was advantageous for a significantly earlier cell colonization, as previously reported for cell-seeded/containing PGA or other cartilage repair material [19,20,22,23,32,41,42,43,44,46,47]. On the other hand, in vivo microfracturing below a cell-free PGA implant is suitable to support cell immigration [19,24].

#### 3.2.2. Local Production of Cartilage Matrix Molecules

Both types of PGA implants (+/− cells) displayed long-term deposition of cartilage matrix molecules, e.g., aggrecan and collagen 2, in line with continuously incrementing gene expression for aggrecan and collagen 2 in both implant types and significantly higher collagen 2 expression in cell-loaded PGA implants at 4 weeks of culture (3225-fold increase vs. 0 weeks). Additionally, locally synthesized, cartilage-specific matrix molecules were successfully retained in the PGA, as suggested by a progressively diminished release of aggrecan and collagen 2 into the supernatant. As mentioned for the cell immigration above, cell-loaded PGA implants sped up and/or augmented the regeneration of cartilage, as underlined by a more extended deposition/presence of aggrecan. This advantage did not only regard the PGA implant but also appeared to stabilize the “host” cartilage ring and cartilage–implant interface (see Figure 7), suggesting an influence of cell-loaded PGA implants on the surrounding model system.

#### 3.2.3. De-Differentiation/Re-Differentiation of CHONDROCYTES

Clear signs of chondrocyte de-differentiation were missing in either cell-free or cell-loaded PGA, since the aggrecan protein content was constant (cell-free) or augmented to a plateau after approximately 10 weeks (cell-loaded), the collagen 1 and 2 content was constant, and the gene expression ratios for aggrecan/collagen 1 (up to 67-fold) and collagen 2/collagen 1 (max. 1227-fold) substantially increased over time. The current model may thus favor extended phenotypic stabilization of the chondrocytes in the PGA (at least up to 12 weeks) despite its progressive resorption and despite the absence of exogenously added chondrogenic mediators ([29]; and references therein). However, partial discrepancies between the mRNA and protein levels for collagen 1 and, to a smaller degree, for aggrecan and collagen 2 may be due to factors such as incomplete translation of mRNA, lack of mechanical loading ([48] and references therein), and/or lack of exogenous stimulation by growth factors or medium components [41,49,50].

#### 3.2.4. Lateral Attachment (Decreased Push-Out Forces)

The push-out forces from the “host” cartilage ring for both types of PGA implants (+/− cells) significantly decreased over time, likely due to the progressive resorption of the PGA [24,32,41]. In line with these findings, PGA loses 50% of its mechanical strength after 7 days and completely degrades in 42 days in PBS ([32,41]; and references therein). Thus, our in vitro model is clearly informative for non-resorbable materials [29,30,31] but may show limitations in assessing the “lateral bonding” of resorbable materials. Since both non-resorbable [51,52,53] and resorbable cartilage implant materials [19,20,22,23,45] are clinically applied, the apparent lack of “lateral bonding” for the present PGA implant in vitro may not be representative of its in vivo performance.

## 4. Materials and Methods

### 4.1. Preparation/Culture of Bovine Cartilage with PGA

Cartilage rings (Ø 6 mm; 96 to 120 replicates from one animal) with an inner defect for implant placement (Ø 2 mm) were aseptically prepared from offal on the day of slaughter from the groove of the femoral trochlea (lateral facets) of German Holstein Friesian Cattle (age 2 years). This was achieved by successively using biopsy punches and a scalpel as previously published ([29,37]; resulting area, height, and volume of the inner defect: 0.0314 cm^2^; 1.3 ± 0.3 mm; 0.0048 cm^3^, respectively; for cell-loaded PGA implants 2 × 10^7^ chondrocytes/cm^3^, i.e., total of 96.000 cells/PGA implant).

For the isolation and expansion of bovine chondrocytes according to the guidelines of the producer (TransTissue Technologies GmbH, Berlin; Germany; for details see [41]), articular cartilage was harvested from the femur condyles of separate adult cattle (age 2 years). In brief, the cartilage was minced and enzymatically digested overnight in a Wheaton^®^ spinner flask (DWK Life Sciences GmbH, Mainz, Germany) under gentle stirring with RPMI 1640 medium (Biochrom GmbH, Berlin, Germany) containing 10% human serum (German Red Cross, Berlin, Germany), 1.5 U/mL collagenase P (Roche, Grenzach-Wyhlen, Germany), 500 U/mL collagenase CLS type II (Biochrom), 50 U/mL hyaluronidase (Sigma-Aldrich, Hamburg, Germany), 100 U/mL penicillin (Biochrom), and 100 mg/mL streptomycin (Biochrom). The resulting cell suspension was centrifuged at 580× *g*, and the cell pellet was washed twice with Hank’s salt solution (Biochrom). The cells were stained with trypan blue (Sigma-Aldrich), counted in a hemacytometer and thereafter seeded into cell culture flasks with an initial density of 2 × 10^5^ cells/cm^2^. The cells were grown in RPMI 1640 supplemented with 10% human serum and antibiotics as described above with a medium exchange every 2 to 3 days. At 80% confluence, the cells were detached using trypsin/EDTA solution (Biochrom) and subcultured with a density of 1 × 10^5^ cells/cm^2^. After three passages, the cells were detached, counted, and resuspended at a density of 2 × 10^7^ cells/mL in fibrinogen (Tissuecol; Baxter, Höchstadt, Germany). The cell/fibrinogen suspension was added to 1 cm^2^ PGA fleeces (Alpha Research GmbH, Berlin, Germany), and a thrombin solution (1:1 *v*/*v* PBS; Tissuecol; Baxter) was subsequently added. Thereafter, PGA scaffolds were placed in the incubator (37 °C, 20 min) to polymerize the fibrinogen.

Circular PGA implants were then punched out of the PGA flecces (+/− bovine chondrocytes) using a 2 mm diameter biopsy punch and aseptically applied into the inner defect of the cartilage rings. The constructs with PGA implants [41] were then fixed in agarose cylinders in 48-well plates, cultured for up to 12 weeks (− cells: n = 5, + cells: n = 6 experimental series) at 37 °C and 5% CO_2_, and analyzed histologically, biochemically, and biomechanically as previously described [29]. Culture supernatants from 1 week each were pooled and preserved for further analysis (−20 °C).

### 4.2. Histology and Immunohistochemistry

Non-cultured or cultured cartilage/PGA hybrids were processed by fixation in phosphate buffered saline (PBS) containing 4% paraformaldehyde and subsequent embedding in paraffin. Conventional histology of cartilage/PGA hybrids was performed in sections (6 µm thickness) stained with either hematoxylin/eosin (HE) or safranin O (to semiquantitatively measure the proteoglycan content). Immunohistological staining for aggrecan, collagen 1, and collagen 2 was executed as published before [29]. Isotype-matched control immunoglobulins always yielded negative results.

### 4.3. Cell Migration Score

Colonization of the PGA implants (cell-free; cell-loaded) was assessed using a published scoring system [29] containing four levels: 0 = implant without cells, 1 = single adherent cells, 2 = several adherent cells, and 3 = cell-layer on the implant.

### 4.4. Safranin O, Collagen 1, Collagen 2, and Aggrecan Score

Stained sections were evaluated using a previously reported semi-quantitative score [29] with 4 levels: 0 = no staining, 1 = weak staining, 2 = moderate staining, and 3 = strong staining.

### 4.5. Quantitative Real-Time Polymerase Chain Reaction

Gene expression of aggrecan, collagen 2, and collagen 1 was analyzed in three different tissue/cell groups. Therefore, RNA was prepared from: (i) the “host” cartilage matrix; (ii) cells on the surface of the cartilage; and (iii) cells on/in PGA implants (+/− cells). RNA isolation, cDNA synthesis, and qRT-PCR (i-cycler PCR system; BioRad, Munich, Germany) were performed as reported before [29] applying established primers (Table 1), the ΔΔ-Ct calculation method, and bovine chondrocyte PCR amplificates as standards for qRT-PCR. Gene expression was normalized to that of the housekeeping gene aldolase. The specificity of the PCR product was verified by melting curve analysis and cycle sequencing.

### 4.6. Protein Preparation

Protein extraction was performed from the three different groups (see above) as previously published [29,30]. In brief, protein was extracted from the “host” cartilage via disintegration with a pair of scissors in 1000 µL of 4 M GuHCL and incubation under rotation for 48 h at 4 °C. Protein extraction from the cells on the cartilage surface was performed using the acetone precipitate of the lysis buffer in line with the instructions of the supplier of the RNeasy mini kit (Qiagen).

### 4.7. Quantification of Glycosaminoglycans

To determine the liberation of sulphated GAG from the cartilage/PGA hybrids and the remaining GAG amount in tissue and surface cells, the DMB Assay was used for quantification [31,54,55]. The supernatants were analyzed as reported [29,30,31].

### 4.8. Enzyme-Linked Immunosorbent Assay

The content of the matrix proteins collagen 1 and 2 in cartilage rings, cells on the cartilage surface, and the supernatant during culture were measured using ELISA-Kits. Supernatants were pooled weekly and analyzed after 0, 4, 8, and 10 weeks of cultivation. The concentrations of matrix proteins were subsequently analyzed using commercial ELISA-Kits (Chondrex™, Redmond, WA, USA; BlueGene, Shanghai, China) [29].

### 4.9. Testing of Push-Out Forces

Implant push-out forces with samples from 0, 4, 8, 10, and 12 weeks in different culture series (in all cases 10 samples) were measured using a static universal test system (Zwicki 1120^®^, Zwick/Roell, Ulm, Germany) at the Department of Orthopedics and Sportsorthopedics, Technische Universität München. Results are reported in Newtons and, to allow a comparison with earlier reports, in kPa (based on dividing the force by the lateral surface of the BNC cylinder (0.8163 mm^2^; [29,30,31]).

### 4.10. Statistical Analysis

Means +/− standard error of the mean were reported. Mann–Whitney U tests and the statistical software SPSS 22.0 were used for statistical analyses (significance level *p* ≤ 0.05).

## 5. Conclusions

Limited proteoglycan release, largely stable tissue integrity and aggrecan/collagen 2 content in the “host” cartilage, and extended phenotypic stabilization of the chondrocytes in cartilage surface and PGA suggest initial cartilage repair in the implant. Significantly accelerated cell immigration and more extended deposition/presence of aggrecan indicate that cell-loading may be advantageous. The apparent lack of “lateral bonding” of the present resorbable PGA implant, however, may require the usage of modified osteochondral “host” cylinders with cartilage defects, but intact subchondral bone plates, in order to obtain results representative of the implant’s in vivo performance.

The current findings may represent a starting point for future clinical trials aimed at improving the broad application of the PGA implant for cartilage defects in different articulating surfaces of knee, ankle, and, possibly, hip joints.

## Figures and Tables

**Table 1:** Primers, product length, and specific amplification conditions for RT-PCR.

Gene	Upstream Primer (5′ 3′)	Downstream Primer (3′ 5′)	Product Length	Annealing Temp.	Melting Temp.
Aggrecan	CAGAGTTCAGTGGGACAGCA	AGACACCCAGCTCTCCTGAA	193	60	84
Collagen 2	CATCTGGTTTGGAGAAACCATC	GCCCAGTTCAGGTCTCTTAG	600	61	83
Collagen 1	AGCCAGCAGATCGAGAACAT	ACACAGGTCTCACCGGTTTC	185	60	86
Aldolase	CACCGGATTGTGGCTCCGGG	CGCCCCCGATGCAGGGATTC	170	58	88

General amplification protocol (40 cycles): initial denaturation for 1.30 min at 95 °C; denaturation for 20 s at 94 °C, specific primer annealing temperature (see above) for 20 s, amplification at 72 °C for 30 s, additional heating step at 84 °C; denaturation for one minute at 95 °C; cooling to 60 °C (holding for 10 s).

## Data Availability

All data reported in this study are available upon request from the corresponding author.

## Supplementary Materials

The following are available online at https://www.mdpi.com/article/10.3390/ijms222111769/s1.
