# Supplementary material for: In Vitro Cartilage Regeneration with a Three-Dimensional Polyglycolic Acid (PGA) Implant in a Bovine Cartilage Punch Model
Source: Int J Mol Sci. 2021 Oct 29;22(21):11769. doi: 10.3390/ijms222111769 (PMC8583898; doi:10.3390/ijms222111769)
Supplement: Supplementary file 1 [file ijms-22-11769-s001.zip › ijms-1421782-supplementary.pdf]

Supplementary Material

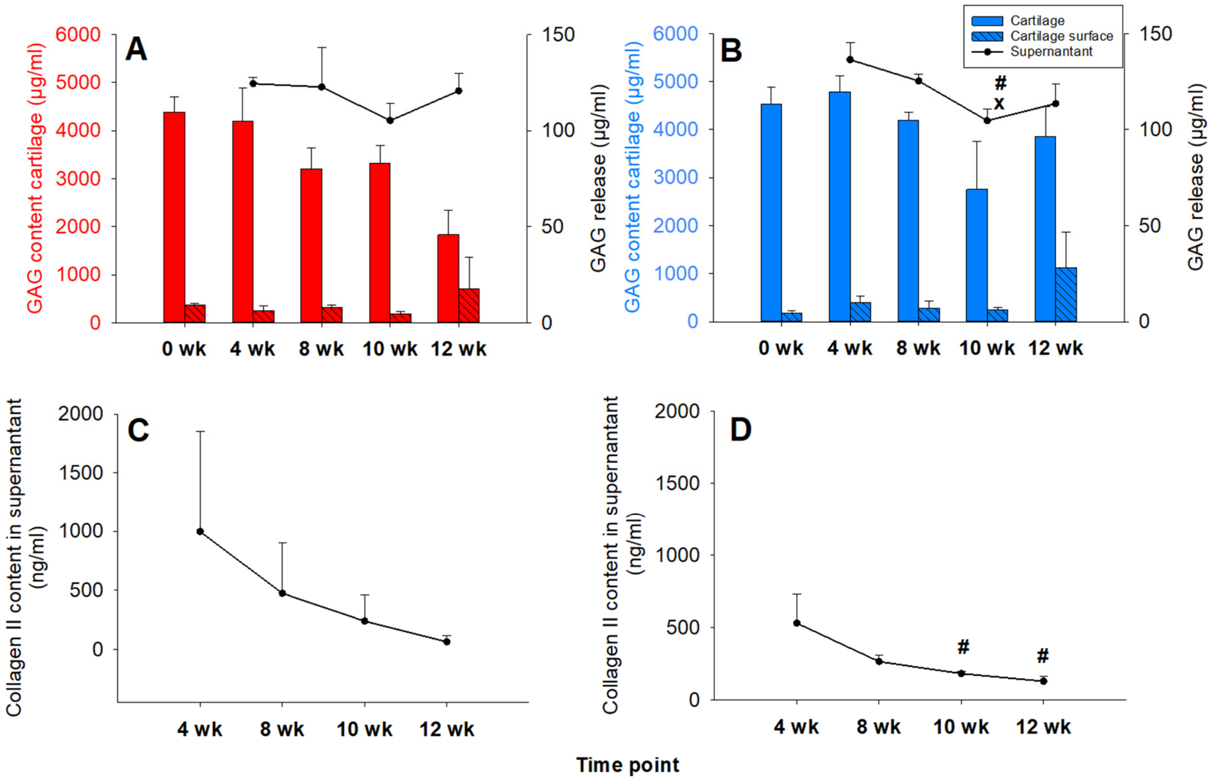

Figure S1. Quantitation of proteoglycan content or liberation (DMB assay) or newly produced collagen 2 (ELISA) in cartilage/PGA-hybrids (cell-free or cell-loaded PGA). Levels of proteoglycans or newly produced collagen 2 were analyzed in fresh and cultured 'host' cartilage (cartilage), cells on the surface of the cartilage (cartilage surface), and supernatant (supernatant), using either cell-free (A for proteoglycan; C for collagen 2) or cell-loaded PGA (B for proteoglycan; D for collagen 2); means ± SEM; symbols show $p \leq 0.05$ versus # 4 weeks; x 8 weeks.
